# Regulatory responses to foodborne illness outbreaks in the United States and their implications for food safety

**DOI:** 10.3389/fnut.2025.1717980

**Published:** 2026-01-16

**Authors:** Grishma Prabhukhot

**Affiliations:** Independent Researcher, Hanover, MD, United States

**Keywords:** food safety, foodborne illnesses, foodborne outbreaks, outbreak investigation, regulatory responses

## Abstract

Foodborne outbreak investigations in the United States are inherently complex. Amid the evolving landscape of policy changes, scientific breakthroughs in disease detection, technological innovations, and globalized trade, there is an urgent need to build a resilient food safety system. This perspective article highlights the role of various regulatory bodies in two of the recent foodborne outbreaks, critically evaluating both the strengths and shortcomings in how these outbreaks were managed. Additionally, the FDA's *New Era of Smarter Food Safety* is discussed in brief to showcase how technologies such as Artificial Intelligence, blockchain, and Internet of Things (IoT) are advancing traceability, outbreak response, and prevention. This article argues that international policy alignment, targeted investment, and inclusive infrastructure development are essential for translating technological advancements into resilient and globally accessible food safety practices.

## 1 Regulatory agencies and their roles

In the United States, approximately 800 foodborne illness outbreaks occur each year (1). Foodborne illnesses raise public health, and safety concerns and burdens the economy. In the year 2023, the cost of foodborne illness was estimated to be $75 billion, wherein deaths were associated with a 56% and chronic outcomes contributed to 31% of the average cost (2). Pathogens associated with highest costs were non-typhoidal *Salmonella* ($17.1 billion) followed by *Campylobacter* ($11.3 billion) (2).

Food Safety oversight in the United States while highly methodical, remains inherently complex mainly due to the involvement of multiple agencies on federal and state level. A foodborne illness outbreak assessment mainly involves three agencies: the Centers for Disease Control and Prevention (CDC), the Food and Drug Administration (FDA), and the U.S. Department of Agriculture (USDA). The U.S. Department of Health and Human Services (HHS) oversees multiple operations and agencies including the FDA and the CDC, whereas the USDA is an independent federal agency. As explained in the Guidelines for Foodborne Disease Outbreak Response by the Council to Improve Foodborne Outbreak Investigations (CIFOR) (3), the CDC identifies, monitors, and can lead the investigations of foodborne outbreaks, the FDA regulates domestic and imported food except meat, poultry, and processed egg products (i.e., frozen, dried, and liquid eggs) with a goal to prevent contamination of food products before distribution. The Food Safety and Inspection Service (FSIS) which is a public health agency within the U.S. Department of Agriculture (USDA) possess the legal authority to regulate meat, poultry, and egg products. Most importantly, while responding to an outbreak, if the product comes under the purview of USDA-FSIS regulated product range, and contains a pathogen, then USDA-FSIS may issue a product recall.

## 2 Recent revisions to surveillance design

Until July 2025, the Foodborne Diseases Active Surveillance Network (FoodNet) conducted surveillance for a total of eight pathogens deemed significant to food safety and public health namely *Campylobacter, Cyclospora, Listeria, Salmonella*, Shiga toxin producing *E. coli* (STEC), *Shigella, Vibrio*, and *Yersinia* infections (4). A reduction in required surveillance by the CDC's FoodNet program was implemented later in the month, with monitoring now focused on two pathogens: *Salmonella* and STEC due to a gap between available funding and the resources needed to continue comprehensive surveillance for all eight pathogens (5–7). However, given that *Listeria monocytogenes* causes an estimated 1,600 illnesses annually in the United States, with case-fatality rates as high as 15–20%, its exclusion from national surveillance represents a substantial concern for public health (8, 9). Although a pivot was deemed necessary in the monitoring program by the FoodNet, the exclusion of certain organisms from its surveillance may reduce the sensitivity of trend detection. Consequently, should there be an uptick in the foodborne illnesses caused due to the other previously monitored pathogens, this step could potentially delay the timely recognition of such increases.

In addition to potentially delaying the detection of emerging trends, the exclusion of certain organisms from FoodNet surveillance may contribute to challenges in aligning regulatory monitoring with food-industry food safety programs. This shift raises questions about whether organisms no longer tracked at the national level will continue to receive appropriate attention within industry monitoring frameworks, possibly influencing prioritization and resource allocation decisions. However, food safety programs within food-processing companies are typically implemented to very high standards. To maintain these standards and mitigate any misalignment, it is important that clear, evidence-based guidance be provided to industry stakeholders. Such guidance should emphasize that despite reduced federal surveillance requirements, continued monitoring of these organisms within company programs remains critical, with proper record-keeping to sustain alignment between surveillance priorities and in-plant food safety practices.

It is important to note that PulseNet, the CDC's national molecular subtyping network, continues to play a crucial role in *Listeria* surveillance. Besides PulseNet, FoodCORE, and Surveillance and Epidemiology Data Resource Integration and Coordination (SEDRIC) platforms are currently ensuring robust monitoring of *Listeria* despite the reduction in active FoodNet surveillance, providing layered and comprehensive pathogen tracking essential for effective foodborne illness control.

## 3 Perspective on outbreak and response coordination

In the United States a foodborne illness is considered an outbreak when two or more people get the same illness from the same contaminated food or drink (10). According to a report published by the WHO in 2015, 600 million foodborne illnesses are caused every year worldwide (11). Foodborne illness outbreak continues to remain a major issue worldwide with thousands affected annually. Given this persistent burden, regulatory measures in the United States have evolved to better protect public health. A key milestone was the enactment of the Food Safety Modernization Act (FSMA) in January 2011, which authorized the FDA to issue mandatory recalls of unsafe food when companies fail to voluntarily take action.

The way a system responds to a food recall can offer insights into its strengths and areas for improvement. Recalls involving meat, leafy greens, seafood etc. require a strong collaboration between the regulatory agencies, public health institutions, and industry. In this section, a perspective on recent key recalls is discussed that have served as milestones in driving improvements to the food-safety system.

### 3.1 Approach for this perspective

#### 3.1.1 Outbreak selection

The two outbreaks discussed in the following sections are: Boars Head deli meats (*Listeria monocytogenes*, July 2024) and McDonalds onions (*E. coli* O157:H7, 2024–2025). These articles were selected due to their recent impact on public health, regulatory significance, and multi-agency involvement. These two outbreaks were also focused on due to their scale and the involvement of major national food brands, which amplified public awareness.

#### 3.1.2 Documents reviewed

- The sources reviewed included:
  - CDC outbreak notices.
  - USDA-FSIS Public Health Information System reports and product recall alerts.
  - FDA outbreak investigation summaries, inspectional observations (Form 483), recall documentation, and CORE Network investigation datasets.
  Supplementary material from company announcements, media advisories, and referenced scientific literature.
- Supplementary Table A Summary: Data Extraction
  - Sources: Supplementary Table A was compiled by extracting investigation summaries from FDA's official dataset and CORE Network records spanning late 2020 through mid-2025.
  - The date range of late 2020 to mid-2025 was selected to ensure up-to-date relevance and capture a full cycle of recent major regulatory and policy changes in food safety surveillance, outbreak investigation, and response systems. This period overlaps with high-profile outbreaks, new federal initiatives such as the FDA's “New Era of Smarter Food Safety,” and shifts in surveillance priorities (e.g., CDC FoodNet program focusing on core pathogens).
  - For the purposes of Supplementary Table A, each outbreak investigation posting was classified as “identified” if the federal notice named at least one specific food or commodity as the vehicle (for example, “leafy greens,” “packaged salad,” seafood,” “mini pastries,” or “pistachio cream”). Outbreaks were classified as “not identified” when no food was specified or when the notice explicitly stated that the food vehicle remained unknown. Broad but food-specific categories such as “leafy greens” or “seafood” were treated as “identified,” whereas entries listing only a pathogen, a general setting, or the term “Not identified” were treated as “not identified.” Using this approach, 65 of 116 outbreaks (56%) were coded as having an identified food vehicle.
  - Only the columns most relevant for the Perspective's comparative analysis of outbreak timing, causative agent, and product attribution were included in Supplementary Table A.
  - Remaining fields, which provide regulatory procedural detail (case count, investigation status such as whether the case is open or closed, regulatory triggers such as Traceability initiated or not), were omitted from Supplementary Table A to focus on the comparative core themes of pathogen, product, and temporal trends. This approach ensures clarity, enables direct comparison of multi-state outbreaks, and was justified to avoid redundancy and over-complexity.
  - Data Extraction Transparency statement: The full dataset (bottom row of years 2020 through 2025) was reviewed to confirm completeness for inclusion in Supplementary Table A.

### 3.2 Boar's Head, a U.S.-based deli meat company

In July 2024, over 7 million pounds of Boar's Head ready-to-eat deli meat products were recalled with CDC reporting 10 deaths in this multi-state outbreak (12). Due to an outbreak being characterized by the occurrence of disease cases above the expected level, the response to it is inherently that of a reactive nature. Although this particular recall was initiated on July 26, 2024, the sample collection spanned over May 29, 2024 to July 12, 2024 (13) thereby indicating that it took around 1 month and 27 days to officially recognize it as an outbreak. In terms of food safety and public health, this timeline reflects a significant delay in detection and response, potentially allowing further spread of the disease.

Details related to various food safety issues at this company are listed in the USDA-FSIS Public Health Information System (14) report. Despite the preventive measures that food processing companies are required to implement, multiple incidents highlight lapses in the enforcement of these safety protocols. A combination of factors such as inactivity from company management and federal agencies contributed to worsening the food safety issues (15). Although violations were identified in 2023, the plant did not face stringent enforcement actions or warning letters from the regulatory authorities. This foodborne outbreak incident underscores that food safety risks can be effectively prevented if the established protocols are rigorously followed and collaborative efforts are maintained at all stages of food processing.

After completion of investigation of this outbreak, the USDA published a review which mentioned that a further examination of the *Listeria* Rule (9 CFR 430.4) is required wherein the FSIS' regulatory approach to *Listeria monocytogenes* will be thoroughly examined (16). The review additionally mentioned that new science-based recommendations will be provided by 2026 on how to make the approach more effective. The report also identified key areas for improvement such as sampling, inspector training, oversight over Talmadge-Aiken federal plants staffed by state inspectors, in addition to the future of the agency's *Listeria* regulatory policy as mentioned above. The future steps outlined in the review underscore the USDA's commitment to continuously improve food safety, implement updates based on scientific advancements in the field, and regulatory policy geared toward strengthening prevention and protection of public health.

A bullet list mentioned below showcases key milestones in the Boar's Head listeriosis outbreak, including first laboratory signal, CDC outbreak announcement, recall initiation and expansion, and subsequent public health updates:

- May 29, 2024: First patient out of a total of 61 patients was identified associated with this outbreak. CDC mentioned that the true number of sick people in this outbreak was likely higher than the number reported because some people recover without medical care and are not tested for *Listeria* (17).
- July 12, 2024: FSIS opened an investigation into a multistate outbreak of listeriosis linked to retail-sliced deli meats. The traceback investigation, done in conjunction with state partners, narrowed the source to M12612 (16).
- July 19, 2024: CDC posts initial multistate listeriosis outbreak notice (outbreak detection/epidemiologic signal) (18).
- July 25, 2024: The Maryland Department of Health confirmed that an unopened package of liverwurst product, produced by M12612 and collected at retail, was positive for Lm. That same day, FSIS suspended inspection of all operations on the production line used to produce liverwurst (19).
- July 26, 2024: Boar's Head initiates recall of ready-to-eat liverwurst products produced at the Jarratt, VA facility (initial recall decision) (20).
- July 30, 2024: Recall expanded to approximately 7 million additional pounds of meat and poultry products produced between May 10 and July 29, 2024 (expanded recall) (20).
- November 21, 2024: CDC announces that the outbreak is over and that implicated products are past shelf life (closure of investigation/public advisory update) (21).

### 3.3 McDonald's restaurant related *E. coli* O157:H7 recall

A recall related to onions served at McDonald's restaurants in 2024–2025 highlighted the importance of persistent challenges in modern outbreak response. This incident particularly emphasized that, despite McDonald's status as a global industry leader and adaptation of modernized and digitized food safety systems, episodic lapses can still occur.

This was also a multi-state outbreak spanning 14 states, resulting in over a 100 illness cases, additionally four cases of hemolytic uremic syndrome (HUS), a severe complication (22). The CDC and FDA identified “likely source of contamination” to be the slivered onions supplied by Taylor Farms (23).

Briefly, the FDA, Colorado Department of Health and Colorado Department of Public Health and Environment conducted a whole-genome sequencing (WGS) to assess whether the *E. coli* strain detected in the onions from Taylor Farm's Colorado facility matched that of people who were facing the foodborne illness (23). The WGS sequence data indicated that although the onions sourced from Taylor Farms tested positive for a non-O157 Shiga toxin-producing *E. coli* (STEC), that strain did not match the outbreak strain or any other clinical illnesses. Since the public summaries do not provide the information related to the depth of sequencing or the comparators, interpretation of these documents is necessarily limited to high-level statements about relatedness. However, the lack of genomic match between the onion isolate and the outbreak strain does not, by itself, refute onions as a vehicle and may reflect lot-to-lot variabilities, timing of sampling relative to production schedules. In this context, the outbreak illustrates how advanced molecular surveillance can identify multiple *E. coli* strains in the production environment, while persistent constraints in traceback records, sampling frames, and environmental data leave residual uncertainty about the precise contamination pathway.

Despite the rapid interagency coordination and collaborative efforts between federal and state regulators, public health officials, and the business entities involved, which enabled the execution of a large-scale recall of Taylor Farms onions supplied to McDonald's, the outbreak was associated with adverse clinical outcomes, including one reported fatality. One of the shortcomings within the response to this outbreak was that the source of contamination was not definitively identified and that Taylor Farms had issued a *voluntary* recall. Although communication between regulatory agencies and the press was swift, disseminating updates across multiple platforms, including news outlets, food safety magazines, consumer advisories, and company websites (24), thereby helping to limit further spread of illness, this incident nonetheless underscored the critical importance of efficient trace-back methodologies.

FDA released form 483 which is a notice of “Inspectional Observations” (25) specifying that the inspection took place around the time period of October 28-November 12th, 2024 (On October 22, 2024, and that Taylor Farms recalled yellow onions that were supplied to McDonald's and other food service customers). Additionally, this form captured key details of the investigation, documenting observations of inadequate sanitation practices, deficiencies in verification records, employee hygiene lapses, and other sanitation-related non-conformities. As these corrective actions were noted at a time period after the actual recall had already begun, this report served as substantial evidence that the preventive measures were not followed by the supplier; Although, inspection itself was not a trigger for the recall. Under current U.S. law, FDA generally relies on firms to implement preventive controls and, in many cases, to initiate recalls voluntarily, whereas USDA-FSIS has different authorities and enforcement tools for meat and poultry. In this outbreak, the Taylor Farms recall proceeded within that voluntary framework, and regulatory agencies operated within existing statutory limits and finite inspection resources. From a policy perspective, more frequent, risk-based audits and inspections of large produce suppliers could potentially prompt earlier corrective actions and reduce the scale or likelihood of similar recalls in the future; however, this should be interpreted as an opinion and not as a proven causal explanation for this specific event. Any such expansion would need to account for statutory authorities, the evidentiary threshold needed to support enforcement actions, and resource constraints that shape how often large facilities can be inspected. Accordingly, the discussion in this Perspective is intended as interpretive commentary on system-level opportunities for improvement rather than a definitive assignment of causality to regulatory “inactivity” in the McDonald's-Taylor Farms outbreak.

Although regulatory bodies have modernized their screening and sampling approaches, these methods primarily serve as verification measures. Sampling strategies have their own set of shortcomings related to detection limits, homogenization of sample, etc. The primary safeguard, however, lies in preventive controls that stop hazards before they reach the food supply. Yet again, this outbreak emphasized that trace-back is a reactive response not preventive.

### 3.4 Summary of recent foodborne outbreak investigations

To share some measurable and actionable steps in near-term, concrete policy actions could be prioritized. The FDA and industry could establish lot-level digital traceability targets for high-risk and high volume commodities such as fresh produce, RTE meats, electronic records that could support rapid trace-backs and trace-forward. The Boars Heads and Taylor Farms outbreaks both highlighted how time needed to reconstruct the distribution patterns directly influenced the scale and duration of recalls. A standardized system which can rapidly integrate the data from different firms and systems could potentially help outbreak-relevant traceability. At a minimum such a dataset could help identify lots across various facilities, supplier and customers, shipment dates and locations, thereby enabling faster linkages between implicated facilities.

More importantly, dedicated training modules and targeted technical assistance should be developed for small- and medium-sized suppliers that support large national brands, with an emphasis on implementing robust digital traceability systems. This is particularly critical because, as illustrated in both cases discussed above, gaps in traceability capabilities at the supplier level can persist even when advanced systems are available downstream, thereby creating substantial bottlenecks in overall food safety performance.

Additionally, the Boars Head and Taylor Farms incidents showed that deficiencies in sanitation and verification may persist even at facilities of large-scale brands irrespective of the resources these companies may have; and that these deficiencies could be detected through an outbreak or intensified inspection, suggesting a role for clearer expectations around audit cadence.

Finally, data management and investigations of foodborne outbreaks is usually conducted by the Coordinated Outbreak Response & Evaluation (CORE) Network established by the FDA in 2011. Data related to the total outbreaks and incidences recorded in recent years by the FDA is presented in Supplementary Table A. This dataset presents foodborne outbreak investigation summaries from the late 2020 to mid-2025, highlighting the causative agents, implicated products, and whether the specific food was identified or not (23). As shown in Supplementary Table A, 65 of 116 outbreaks in the 2020–2025 dataset (56%) had at least one specific food or commodity identified as the vehicle, while 51 outbreaks (44%) remained without an identified product by the end of the investigation. This near-even split indicates limited progress over time in enhancing traceability and source attribution. This trend also highlights the challenges in outbreak investigation surveillance systems, the complex nature of investigations wherein multiple regulatory agencies are responsible for oversight. Overall, the data underscores the continued need for improved harmonization and efficiency in outbreak investigation frameworks.

Historically, large multistate outbreaks associated with ready-to-eat meats and produce have exposed recurring weaknesses in sanitation, environmental monitoring, and traceability, prompting incremental regulatory change. Earlier outbreaks linked to meats and fresh produce led to stronger emphasis on environmental sampling, pathogen control, and traceback expectations, but still relied heavily on paper-based records and slower epidemiologic linkages. In contrast, the recent Boar's Head and McDonald's—Taylor Farms outbreaks occurred in a landscape shaped by FSMA preventive controls, enhanced environmental monitoring expectations, and initiatives such as FDA's New Era of Smarter Food Safety, which promote digital traceability and more integrated, data-driven investigations. Compared with past recalls, these two recent cases illustrate both progress and persistent gaps. On one hand, whole genome sequencing, centralized recall-tracking networks, and multi-agency coordination enabled relatively rapid detection, case linking, and multistate communication, features that were far less mature in earlier outbreaks. On the other hand, delays in recognizing patterns seen in earlier outbreaks as well-remained evident. Taken together, this comparison suggests that U.S. regulatory responses have evolved toward more sophisticated tools and frameworks, but that structural issues in traceability, supplier oversight, and outbreak attribution continue to limit the full benefits of these advances.

### 3.5 Digitization and future directions

The food industry has long stood at a unique intersection, comprising contributors from diverse literacy levels as well as varied economic, cultural, and geopolitical backgrounds. Farmers in underdeveloped and developing regions often face resource limitations that hinder their ability to digitize operations and adopt advanced practices, creating disparities when compared to large food corporations that possess the infrastructure and means to ensure food safety.

Modernization efforts are undeniably appealing, having demonstrated both practicality and effectiveness. Specifically an initiative by the FDA called *The New Era of Smarter Food* (26) is a commendable step toward utilizing the capabilities of technology to solve the issues faced by consumers and stakeholders in this modern world of food consumption and processing. The core elements of this initiative as mentioned in the blueprint document include:

1. Tech-enabled Traceability
2. Smarter Tools and Approaches for Prevention and Outbreak Response
3. New Business Models and Retail Modernization
4. Food Safety Culture

Utilization of predictive modeling, artificial intelligence, blockchain technology, sensor technology, and the Internet of Things are undoubtedly improving the traceability, food fraud, trace-forward and trace-backs, outbreak responses, accelerated recalls to name a few. However, in order to harness these solutions to their optimum capabilities, the food industry and regulatory bodies across the globe need to actively take steps toward improving digital literacy, and build infrastructure capable of handling complex data especially in regions that are under-developed and support the growth of technology in growing economies given the dependence of food supply chain on a complex geographical network.

The question of who holds responsibility for addressing disparities in digital literacy and ensuring equitable access to modern platforms is more complex than can be fully examined within the scope of this article. Nonetheless, it is imperative that stakeholders, including government agencies and commercial enterprises, take active measures to ensure that primary producers benefit from these advancements. Moreover, international policy alignment to ensure more investment in digitization of agricultural practices across the countries engaged in agricultural trade and to build an inclusive infrastructure to support food safety would help lower the barriers to knowledge dissemination.

## Data Availability

The original contributions presented in the study are included in the article/Supplementary material, further inquiries can be directed to the corresponding author.
