# Supplementary material for: Regulatory responses to foodborne illness outbreaks in the United States and their implications for food safety
Source: Front Nutr. 2026 Jan 16;12:1717980. doi: 10.3389/fnut.2025.1717980 (PMC12855058; doi:10.3389/fnut.2025.1717980)
Supplement: Supplementary file 1 [file Table_1.docx]

Regulatory Responses to Foodborne Illness Outbreaks and Their Implications for Food Safety

Grishma Prabhukhot*

Independent Researcher, Maryland, USA

*** Correspondence:**Grishma Prabhukhot
grishmasprabhukhot@gmail.com

# Supplementary Material

**Table A**: Recent Foodborne illnesses and products linked to the incidents.

| **Date Posted** | **Pathogen** | **Product(s) linked to illnesses (if any)** |
| --- | --- | --- |
| 12/23/2020 | *Salmonella* | Not identified |
| 11/18/2020 | *E. coli* O157:H7 | Leafy Greens |
| 11/18/2020 | *E. coli* O157:H7 | Not identified |
| 11/18/2020 | *E. coli* O157:H7 | Not identified |
| 11/18/2020 | *Salmonella* | Not identified |
| 11/18/2020 | *Salmonella* | Not identified |
| 11/18/2020 | *Salmonella* | Not identified |
| 11/18/2020 | *Salmonella* | Not identified |
| 12/29/2021 | *E. coli* O157:H7 | Packaged Salad |
| 12/20/2021 | *Listeria monocytogenes* | Packaged Salad |
| 12/15/2021 | *Listeria monocytogenes* | Packaged Salad |
| 11/24/2021 | *Salmonella* | Cut Cantaloupe |
| 11/17/2021 | *E. coli* O157:H7 | Spinach |
| 10/6/2021 | *Listeria monocytogenes* | Not identified |
| 9/15/2021 | *Salmonella* | Red, Yellow and White Onions |
| 9/15/2021 | *Salmonella* | Seafood |
| 8/11/2021 | *Cyclospora* | Not identified |
| 7/14/2021 | *Salmonella* | Salad greens |
| 7/14/2021 | *Cyclospora* | Not identified |
| 7/14/2021 | *E. coli* O121 | Cake Mix |
| 6/9/2021 | *Salmonella* | Shrimp |
| 4/21/2021 | *Salmonella* | Cashew Brie |
| 4/28/2021 | *E. coli* O145:H28 | Not identified |
| 3/17/2021 | Acute Non-viral Hepatitis | Alkaline Bottled Water |
| 2/17/2021 | *Listeria monocytogenes* | Hispanic-style fresh and soft cheese |
| 2/17/2021 | *E. coli* O157:H7 | Not identified |
| 1/13/2021 | *Salmonella* | Not identified |
| 12/28/2022 | *Salmonella* | Alfalfa sprouts |
| 11/16/2022 | *Salmonella* | Not identified |
| 11/9/2022 | *Listeria monocytogenes* | Enoki mushrooms |
| 10/24/2022 | *E. coli* O157:H7 | Not identified |
| 10/12/2022 | *E. coli* O121:H19 | Frozen Falafel |
| 9/28/2022 | *Salmonella* | Seafood |
| 9/14/2022 | *Listeria monocytogenes* | Brie and camembert cheese |
| 8/31/2022 | *Salmonella* | Not identified |
| 8/17/2022 | *Salmonella* | Cantaloupe |
| 8/17/2022 | *Salmonella* | Not identified |
| 8/17/2022 | *E. coli* O157:H7 | Not identified |
| 8/3/2022 | *Cyclospora* | Not identified |
| 7/27/2022 | *Cyclospora* | Not identified |
| 6/29/2022 | Not identified | Frozen food |
| 6/22/2022 | *Salmonella* | Not identified |
| 6/22/2022 | *Salmonella* | Not identified |
| 6/15/2022 | *Listeria monocytogenes* | Not identified |
| 6/8/2022 | *E. coli* O157:H7 | Not identified |
| 6/1/2022 | Hepatitis A virus | Strawberries |
| 5/25/2022 | *Salmonella* | Peanut Butter |
| 4/20/2022 | Not identified | Dry Cereal |
| 4/13/2022 | *Listeria monocytogenes* | Ice Cream |
| 3/30/2022 | Not identified | Meal Replacement Drink |
| 3/16/2022 | *Salmonella* | Not identified |
| 2/17/2022 | *Cronobacter* | Powdered Infant Formula |
| 2/9/2022 | *Listeria monocytogenes* | Not identified |
| 2/2/2022 | *E. coli* O143:H26 | Not identified |
| 1/10/2022 | *E. coli* O121:H19 | Romaine |
| 12/6/2023 | *Listeria monocytogenes* | Not identified |
| 11/22/2023 | *Salmonella* | Not identified |
| 11/22/2023 | *Salmonella* | Cantaloupe |
| 11/15/2023 | *Listeria monocytogenes* | Peaches, Plums, and Nectarines |
| 11/15/2023 | *E. coli* O103 | Not identified |
| 11/1/2023 | *E. coli* O121:H19 | Not identified |
| 10/4/2023 | *Salmonella* | Onions |
| 8/30/2023 | *Salmonella* | Watermelon and cantaloupe |
| 8/30/2023 | *Cryptosporidium* | Not identified |
| 8/9/2023 | *Listeria monocytogenes* | Ice Cream |
| 7/26/2023 | *Cyclospora* | Not Identified |
| 7/26/2023 | *E. coli* O26 | Not Identified |
| 7/12/2023 | *E. coli* O157:H7 | Not Identified |
| 7/6/2023 | *Cyclospora* | Not Identified |
| 6/14/2023 | *Cyclospora* | Not Identified |
| 6/14/2023 | *Salmonella* | Pico de gallo |
| 5/24/2023 | *Salmonella* | Raw Cookie dough |
| 5/24/2023 | *Cyclospora* | Broccoli |
| 4/26/2023 | Not identified | Morel mushroom |
| 3/29/2023 | *Salmonella* | Raw flour |
| 3/8/2023 | *Salmonella* | Not Identified |
| 3/1/2023 | Hepatitis Virus A | Frozen strawberries |
| 2/15/2023 | *Listeria monocytogenes* | Leafy Greens |
| 12/26/2024 | *Listeria monocytogenes* | Frozen Shakes |
| 12/11/2024 | *E. coli* O145:H28 | Not Identified |
| 12/4/2024 | *E. coli* O157:H7 | Romaine Lettuce |
| 11/27/2024 | *Salmonella* | Imported Cucumbers |
| 11/6/2024 | *E. coli* O121:H19 | Organic carrots |
| 10/30/2024 | *E. coli* O26:H11 | Sprouts |
| 10/23/2024 | *E. coli* O157:H7 | Onions |
| 11/14/2024 | *E. coli* O157:H7 | Not Identified |
| 10/23/2024 | *Listeria monocytogenes* | Alfalfa sprouts |
| 10/17/2024 | *Salmonella* | Not Identified |
| 10/9/2024 | *Salmonella* | Not Identified |
| 9/4/2024 | *Salmonella* | Eggs |
| 8/28/2024 | *E. coli* O157:H7 | Spinach |
| 8/21/2024 | *Listeria monocytogenes* | Not Identified |
| 8/7/2024 | *Salmonella* | Not Identified |
| 8/7/2024 | *Cyclospora* | Shrimp salad |
| 7/31/2024 | *Cyclospora* | Imported parsley |
| 7/10/2024 | *Salmonella* | Mangoes |
| 6/19/2024 | *Salmonella* | Jalapeno pepper |
| 6/12/2024 | Not identified | Chocolate, gummies and cones |
| 5/22/2024 | *Salmonella* | Cucumbers |
| 5/22/2024 | *Listeria monocytogenes* | Bagged salad mix |
| 4/17/2024 | *E. coli* O157:H7 | Organic walnuts |
| 4/10/2024 | *Salmonella* | Organic fresh basil |
| 2/20/2024 | *E. coli* O157:H7 | Raw cheddar cheese |
| 1/24/2024 | *Listeria monocytogenes* | Queso Fresco and Cotija Cheese |
| 7/16/2025 | *Salmonella* | Not Identified |
| 6/13/2025 | *Salmonella* | Pistachio Cream |
| 5/14/2025 | *E. coli* O145:H28 | Not Identified |
| 5/14/2025 | *Salmonella* | Cucumbers |
| 5/7/2025 | Hepatitis A virus | Not Identified |
| 4/30/2025 | *Listeria monocytogenes* | Ready-to-Eat foods |
| 4/23/2025 | *Salmonella* | Eggs |
| 3/13/2025 | *Listeria monocytogenes* | Not Identified |
| 3/5/2025 | *Listeria monocytogenes* | Not Identified |
| 2/20/2025 | *Salmonella* | Not Identified |
| 2/5/2025 | *Salmonella* | Mini Pasteries |
| 1/15/2025 | *Listeria monocytogenes* | Not Identified |
